# Impact of Oral Probiotics in Amelioration of Immunological and Inflammatory Responses on Experimentally Induced Acute Diverticulitis

**DOI:** 10.1007/s12602-022-09969-7

**Published:** 2022-07-15

**Authors:** Maha G. Soliman, Hanaa A. Mansour, Wedad A. Hassan, Eman Shawky

**Affiliations:** 1 Department of Zoology, Faculty of Science, Al-Azhar University, Cairo, Egypt; 2 Department of Pharmacology, National Organization for Drug Control and Research (NODCAR), Giza, Egypt

**Keywords:** Probiotics, Inflammatory responses, Lipopolysaccharide, Acute diverticulitis

## Abstract

Acute diverticulitis is inflammation of a colon diverticulum; it represents a major cause of morbidity and mortality. The alteration of gut microbiota contributes to the promotion of inflammation and the development of acute diverticulitis disease. Probiotics can modify the gut microbiota, so they are considered a promising option for managing diverticulitis disease. This study aimed to investigate the potential protective effect of probiotics, alone or in combination with amoxicillin, on the experimentally induced model of acute diverticulitis disease. Forty-two rats were divided into seven groups as follows: control group: received water and food only; DSS group: received 3% dextran sulfate sodium (DSS) daily for 7 days; LPS group: injected with lipopolysaccharide (LPS) enema at the dose of (4 mg/kg); probiotics group: treated with probiotics (*Lactobacillus acidophilus* and *Bifidobacterium lactis*) each of which (4 × 10^8^ CFU suspended in 2 ml distilled water) orally for 7 days; DSS/LPS group: received DSS and LPS; DSS/LPS treated with probiotics group; DSS/LPS treated with probiotics and amoxicillin group. The results revealed that both treatments (probiotics and probiotics-amoxicillin) attenuated DSS/LPS-induced diverticulitis, by restoring the colonic antioxidant status, ameliorating inflammation (significantly reduced TNF-α, interleukins, interferon-γ, myeloperoxidase activity, and C-reactive protein), decreasing apoptosis (through downregulating caspase-3), and reduction of the colon aerobic bacterial count. These probiotic strains were effective in preventing the development of the experimentally induced acute diverticulitis through the anti-inflammatory and immunomodulatory effects and have affected gut microbiota, so they can be considered a potential option in treating acute diverticulitis disease.

## Introduction

Colonic acute diverticulitis is a gastrointestinal disease that is associated with a high rate of morbidity, as well as health care costs. Obesity, diet, and physical activity have been demonstrated as risk factors for acute diverticulitis disease. Also, the genetic factors, as well as alterations in the colon neuro-musculature, participate in the development of acute diverticulitis [1]. Furthermore, it was hypothesized that diverticular disease results from the deficiency of dietary fibers. The hypothesis is that colons in areas of high fiber intake are large-bore compared to areas of low-fiber intake. The pressure required to distend the colon, according to Laplace’s law, is greater where the radius is small. According to this law, the passage of fecal material through a narrow colon requires a greater luminal pressure, which in turn leads to the formation of a colon diverticulum [2]. The colon diverticulum is formed as a consequence of herniation of colon mucosa and submucosa, through the perivascular connective tissue sheath, which surrounds the intramural vasa recta [3].

The physiopathology of acute diverticulitis involves the interactions of individual predisposition and environmental factors, such as colonic stasis, obstruction of the diverticula, and alterations of the gut microbiota [4]. Alteration of the gut microbiota, as well as bacterial overgrowth, occurs due to a prolonged colonic transit and retention of the fecal material within diverticula, which in turn triggers intestinal inflammation by impairing mucosal barrier function and upregulating inflammatory cytokine release [5]. Additionally, gut microbiota may favor diverticular disease development through a pathological enhancement of colonic gas production as well as by increasing the occurrence of inflammation around and inside diverticular mucosa [6]. Consequently, the manipulation of the gut microbiota composition through the use of antibiotics or probiotics has recently been proposed as a further therapeutic option for this condition. Probiotics can be used in the treatment of diverticular disease in order to restore a healthy colonic microenvironment [7] since they have the ability to modify the localized and persistent inflammation in patients who are between acute bouts of diverticulitis. Also, probiotics can act on symptom development, in individuals affected by the uncomplicated diverticular disease [8].

Different mechanisms of action of probiotics have been discussed, such as inhibition of adherence and translocations of pathogens, competitive metabolic interactions with pro-inflammatory bacterial strains, and downregulation of pro-inflammatory cytokines. Also, probiotics can improve mucosal defense at the levels of immune and epithelial function [9]. Moreover, Fric and Zavoral [10] have recently shown the effectiveness of probiotics in treating uncomplicated acute diverticulitis disease. Some specific strains of probiotics maintain adequate bacterial colonization of the gastrointestinal tract and inhibit colonic bacterial overgrowth and pathogen metabolism. In this way, they can increase the anti-inflammatory effects and enhance anti-infection defenses [7]. *Lactobacillus* and *Bifidobacterium* are two types of probiotics that are extensively observed in the human intestine. The previous studies demonstrated that these probiotics have anti‑inflammatory and immunomodulatory activities. Certain *Lactobacillus* strains can upregulate the expression of mucin 3 and enhance the intestinal mucus layer, thereby improving colonic barrier function [11]. Furthermore, it was observed that *Bifidobacterium adolescentis*, *Lactobacillus*, *Phascolarctobacterium*, and *Akkermansia muciniphila* were reduced in patients with intestinal inflammation. Interestingly, when they were present, they reduced the inflammation, particularly through acting on C-reactive protein (CRP), IL-6, and tumor necrosis factor (TNF)-α [12]. Also, Lactobacilli have been demonstrated to reduce symptomatic uncomplicated diverticular disease, particularly in reducing bloating and abdominal pain, while *Lactobacillus acidophilus*, *Bifidobacterium lactis*, and *Lactobacillus salivarius* were effective in the management of acute diverticulitis disease [13]. This study is the first experimental model for acute diverticulitis disease and aimed to investigate the potential protective effect of probiotics, alone or in combination with amoxicillin, against DSS/LPS-induced acute diverticulitis by using 3% dextran sulfate sodium (DSS) daily for 7 days and lipopolysaccharide (LPS) enema (4 mg/kg).

## Materials and Methods

### Chemicals and Drugs

Dextran sulfate sodium (DSS) salt (molecular weight: 40,000) was purchased from Alfa Aesar (ThermoFisher, Kandel, GmbH, Germany); lipopolysaccharide (LPS) was purchased from Sigma-Aldrich (St. Louis, MO, USA); and probiotics (*Lactobacillus acidophilus* and *Bifidobacterium lactis*) were purchased from PharmaCare Europe Ltd (West Sussex, RH10 9NQ, UK). Amoxicillin antibiotic was purchased from the Egyptian International Pharmaceutical Industries Company (Industrial Area, Egypt). DSS and LPS were dissolved in distilled water. The probiotics pellets were dilacerated using a ceramic mortar and pestle, then suspended in distilled water, and shaken well before treatment. Amoxicillin was suspended in distilled water and shaken well before treatment.

### Experimental Animals

Male albino rats (Sprague Dawley), weighing 150–160 g, obtained from the animal house of the National Organization for Drug Control and Research (NODCAR, Giza, Egypt), were used in the present study. Animals were housed for at least 1 week in the laboratory room before testing under controlled environmental conditions: constant temperature (25 ± 2 °C), humidity (60 ± 10%), and alternating 12 hrs light/dark cycles. Standard pellet diet and water were allowed ad libitum. All animal procedures were performed following the Institutional Ethics Committee and under the recommendations for the proper care and use of laboratory animals. The study was approved by the Ethics Committee for Animal Experimentation of Cairo University. Unnecessary disturbance of animals was avoided. Animals were treated gently; squeezing, pressure, and tough maneuvers were avoided.

### Experimental Design

Animals were randomly divided into seven groups, with 6 rats in each group, for a study period of 7 days, as follows: group I, control group: rats only received water and food (no treatment); group II, DSS group: rats have received 3% DSS solution, added to their drinking water, daily for 7 days; group III, LPS group: rats were injected with LPS enema, by a catheter, at the dose of (4 mg/kg), 48 hrs before sacrificing them at the end of the experiment; group IV, probiotics-treated group: rats were treated with probiotics (*Lactobacillus acidophilus* and *Bifidobacterium lactis*) each of which (4 × 10^8^ CFU suspended in 2 ml distilled water) orally, once daily for 7 days; group V, DSS/LPS group: in which acute diverticulitis was induced, rats have received DSS and LPS enema, similar to the ways mentioned above, in DSS and LPS groups; group VI, DSS/LPS-probiotics-treated group: rats were received DSS, LPS, and probiotics; at the doses and ways mentioned in DSS, LPS, and probiotics groups; group VII, DSS/LPS-probiotics-amoxicillin-treated group: rats were received DSS, LPS, probiotics (at the doses and ways mentioned in DSS, LPS, and probiotics groups), as well as amoxicillin (0.162 g suspended in 2 ml distilled water/rat, by oral gavage, once daily for 7 days).

### Induction of Acute Diverticulitis

Acute diverticulitis was experimentally induced by adding 3% weight/volume of dextran sulfate sodium (DSS), dissolved in distilled water, to the rats drinking water, daily for 7 days [14].

Furthermore, the rats were injected with LPS enema, by a Nelaton catheter 8 FG, 48 hrs before sacrificing the rats, at the end of the experiment, where local immune reaction by LPS seems to play an important role in the perpetuation of experimental diverticulitis [15] and aggravating colon inflammation [16].

### Blood Sampling and Colon Tissue Preparation

At the completion of the days of the experiment, rats were sacrificed to enable blood and tissue collection. The serum was separated from the rats’ blood by centrifugation of blood at 4000 R.P.M. for 10 min, divided into aliquots, and stored at –70 °C until used for analysis. Colons were excised from the ileocecal junction to the anus. Part of the colon from each rat (unique for all rats, 8 cm away from the anus) was separated and fixed in 10% formaldehyde for later use in the immunohistochemical analysis. Portions of 1 cm of the distal colon were collected, emptied from the contents, and weighed for use in the aerobic bacterial viable count. The rest of the colons were wrapped in aluminum foil and kept frozen at –70 °C until used for analysis.

### Estimation of Oxidant/Antioxidant Status in the Colon Tissue

The colon reduced glutathione (GSH), nitric oxide (NO), and malondialdehyde (MDA) concentrations were measured colorimetrically using a Biodiagnostic kit (Cairo, Egypt).

### Determination of Myeloperoxidase (MPO) Activity in the Colon Tissue

MPO activity was evaluated using a kinetic colorimetric technique described by Bradley et al. [17]. The colon tissues’ homogenates were subjected to three cycles of freezing and thawing (− 70 °C/37 °C). Then, the homogenates were centrifuged at 10,000 rpm at 4 °C for 15 min. Fifty microliters of the supernatant was separated for use in the MPO assay. MPO activity was evaluated by adding and incubating 50 μl of the supernatant for 5 min at 37 °C to 2.4 ml of 50 mM potassium phosphate buffer (K_2_HPO_4_), PH 6.0, containing 0.167 mg/ml of ortho-dianisidine dihydrochloride, and 4.0 μl of 30% hydrogen peroxide (H_2_O_2_). Ortho-dianisidine is oxidized by MPO in the presence of H_2_O_2_ and produces a yellowish-orange product that can be absorbed at 460 nm. One unit of MPO activity is defined as that required, to degrade 1 μmol of H_2_O_2_ per minute at 25 °C. The results were expressed as (U/g of tissue).

### Determination of Serum C‐Reactive Protein

Serum CRP was measured using a Spinreact® kit (Girona, Spain). The test is based on the principle of latex agglutination.

### Measurement of TNF-α, IFN-γ, IL-1β, and IL-18 in the Colon Tissue

The levels of TNF-α, IFN-γ, IL-1β, and IL-18 were measured, in the colon tissue, using an enzyme-linked immunosorbent assay (ELISA) kit (MyBiosource, Inc., San Diego, USA), according to the manufacturer’s instructions.

### Immunohistochemical Detection of Caspase-3

Caspase-3 level in the colon tissues was examined according to Martín-Burriel et al. [18]. Sections from the colon tissues were incubated with primary antibodies against caspase-3 (1:100 dilution) (Santa Cruz Biotechnology Inc., Dallas, TX, USA). The immune reaction was visualized using diaminobenzidine tetrachloride (DAB, Sigma Chemical Co., St. Louis, MO, USA). Quantification of caspase-3 was estimated by measuring the area % expression from 5 randomly chosen fields in each section and averaged using image analysis software (Image J, version 1.46a, NIH, Bethesda, MD, USA).

### Aerobic Bacterial Viable Count

Portions of 1 cm of the distal colon were collected aseptically, transported to a sterile Petri dish, emptied and washed in sterile saline, and then weighed. The tissue specimens obtained from each rat were separately mashed and dilacerated using sterile ceramic mortar and pestle. Then, the homogenate was flashed aseptically in 9 ml of Ringer’s solution and mixed thoroughly. The diluted homogenate was transferred to a clean sterile 15-ml tube. The plate culture counts were determined by standard plate technique [19]. Next, 1 ml of the homogenate was diluted to one-tenth of its original concentration (1:10). The specimens were subjected to a series of tenfold dilutions in sterile nutrient broth, and triplicate samples of 100 μl from each dilution were plated on nutrient agar. Plates were incubated aerobically at 37 °C for 18 h. The aerobic bacterial count was expressed as colony-forming units (CFU) per gram of tissue [20, 21]. To meet the criteria for statistical accuracy of bacterial numbers in given specimen samples, they were plated in triplicate, and CFU was counted only from plates yielding between 20 and 200 visible colonies [22].

### Statistical Analysis

Statistical differences between groups were computed by one-way analysis of variance (ANOVA) followed by Tukey–Kramer test for multiple comparisons. The results were analyzed using IBM SPSS statistics software (version 28.0). *p* values of < 0.05 were considered as the minimum level of significance.

## Results

### Effect of Probiotics and Probiotics-Amoxicillin on GSH, MDA, and NO Levels in the Colon Tissue

The level of reduced glutathione (GSH) was significantly diminished (*p* < 0.001) in DSS, LPS, and DSS/LPS groups, compared to the control group. Otherwise, treatment with probiotics, in the rats subjected to DSS/LPS, significantly restored the colon GSH level to the normal values (*p* < 0·001), compared to DSS/LPS group (Table 1), while the treatment with probiotics-amoxicillin significantly increased the colon GSH level (*p* < 0·05), compared to DSS/LPS group (Table 1). Malondialdehyde (MDA) levels were significantly elevated in DSS and DSS/LPS groups (*p* < 0·001) and (*p* < 0·01) in LPS group, compared to the control group. On the other hand, treatment with probiotics or probiotics-amoxicillin caused a significant decrease in MDA level (*p* < 0.001; *p* < 0.01, respectively), compared to DSS/LPS group. Nitric oxide (NO) concentration significantly increased (*p* < 0.05) in DSS group and (*p* < 0.01) in DSS/LPS group (*p* < 0.01), when compared to the control group. Only treatment with probiotics decreased NO level (*p* < 0.01), compared to DSS/LPS group (Table 1).

**Table 1:** Effect of probiotics and probiotics-amoxicillin on colon tissue content of GSH, MDA, and NO levels in the different studied groups

**Groups**	**Parameters**		
	**GSH (µMol/g)**	**MDA (µMol/g)**	**NO (µMol/g)**
**Control**	4.51^c^	3200^c^	226.4^b^
**DSS**	3.61^b^	6247^a^	383.6^a^
**LPS**	3.34^b^	5660^b^	266.9^b^
**Probiotics**	3.98^b^	3377^c^	207.0^b^
**DSS/LPS**	3.15^a^	7691^a^	421.3^c^
**DSS/LPS-probiotics**	4.31^c^	3503^c^	247.9^b^
**DSS/LPS-probiotics-amoxicillin**	3.77^b^	5344^b^	308.7^a^
**SEM**	0.076	280.95	14.382
***p*** **value**	< 0.001	< 0.001	< 0.001

Means in the same column, within each parameter, having different superscripts, are significantly different

*GSH* reduced glutathione, *MDA* malondialdehyde, *NO* nitric oxide, *SEM *standard error of mean

### Effect of Probiotics and Probiotics-Amoxicillin on Colon MPO Activity and Serum CRP Levels

**Fig. 1:**
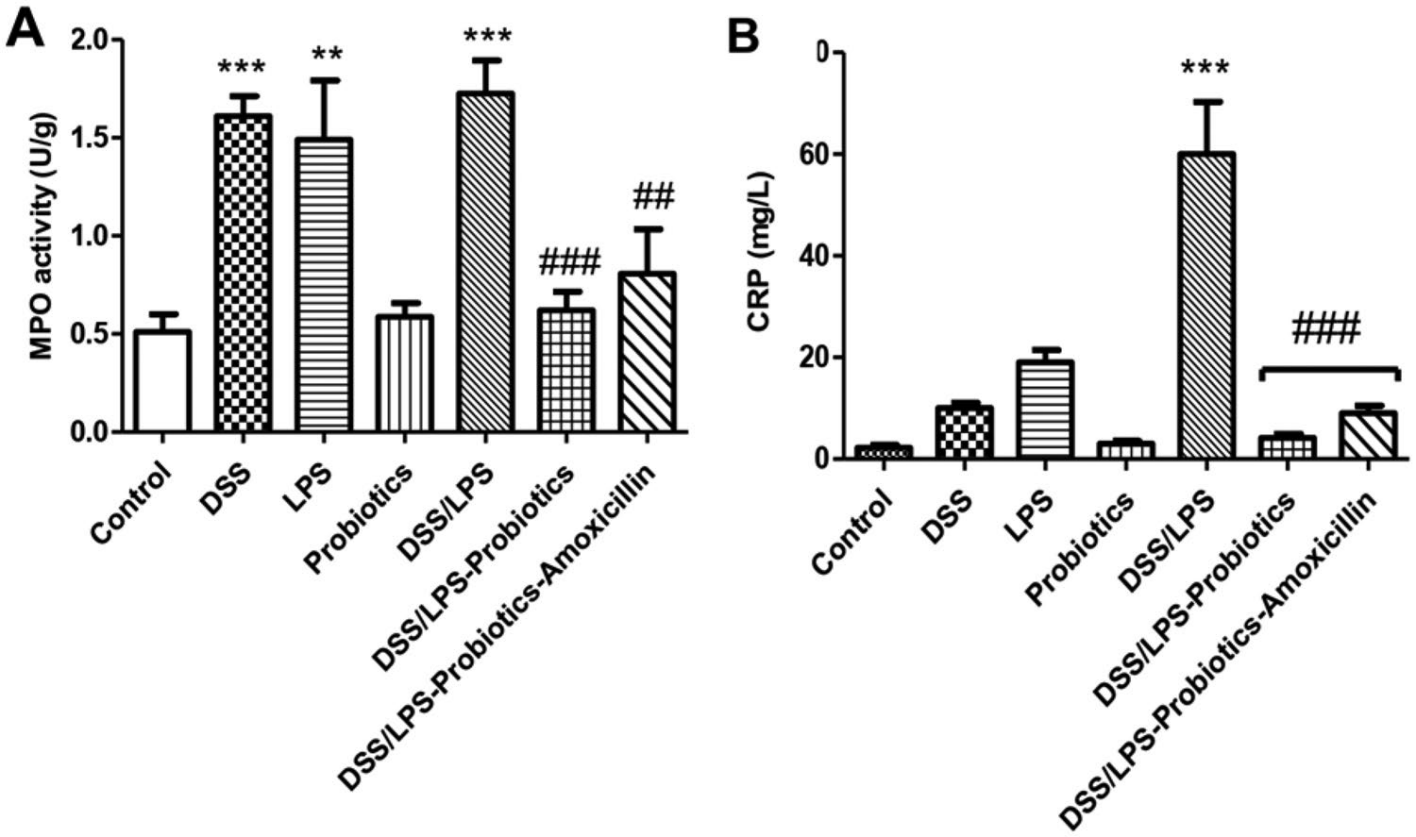
Effect of probiotics and probiotics-amoxicillin on DSS/LPS-induced acute diverticulitis. **A** MPO (myeloperoxidase) activity, in the colon tissue, **B** serum CRP (C-reactive protein) concentration. Statistical analysis was performed using one-way ANOVA followed by the Tukey–Kramer test. ^**^ and ^***^ represent significant differences from the control group (^**^p < 0.01, ^***^p < 0.001). ^##^ and ^###^ represent significant differences from DSS/LPS group ( ^##^*p* < 0.01, and ^###^*p* < 0.001)

As shown in Fig. 1, MPO activity was significantly elevated (*p* < 0.001) in DSS and DSS/LPS groups and (*p* < 0.01) in LPS group, compared to the control group. Moreover, treatment with probiotics (with or without amoxicillin) retrieved MPO activities to the normal values.

Administration of DSS or injection with LPS enema showed a non-significant increase (*p* > 0.05) in serum CRP level compared to the control group. Contrariwise, serum CRP level was significantly increased (*p* < 0.001) in the DSS/LPS group, compared to the control group. On treatment with probiotics or probiotics-amoxicillin, in DSS/LPS-manipulated rats, the levels of serum CRP were normalized and became significantly lower than DSS/LPS group at *p* < 0.001.

### Effect of Probiotics and Probiotics-Amoxicillin on TNF-α, IL-1β, IFN-γ, and IL-18 Levels in the Colon Tissue

As shown in Table 2, administration of DSS or DSS/LPS resulted in a marked increase in the colonic TNF-α, IL-1β, IFN-γ, and IL-18 levels (*p* < 0.001), compared to the control group. LPS enema only resulted in raising TNF-α level (*p* < 0.01) compared to the control group. Either treatment of rats with probiotics or probiotics-amoxicillin decreased the levels of TNF-α, IL-1β, IFN-γ, and IL-18 significantly (*p* < 0.001), compared to DSS/LPS group.

**Table 2:** Effect of probiotics and probiotics-amoxicillin on colonic levels of TNF-α, IL-1β, IFN-γ, and IL-18 in the different studied groups

**Groups**	**Parameters**			
	**TNF-α (ng/g)**	**IL-1β (ng/g)**	**IFN-γ (pg/g)**	**IL-18 (ng/g)**
**Control**	12.31^c^	13.08^c^	12.4^b^	13.41^a^
**DSS**	42.24^a^	39.16^a^	37.29^a^	22.24^b^
**LPS**	19.56^b^	18.68^c^	17.05^b^	17.61^a^
**Probiotics**	13.83^c^	13.06^c^	12.18^b^	12.98^a^
**DSS/LPS**	44.35^a^	54.55^a^	40.1^a^	46.92^c^
**DSS/LPS-probiotics**	27.82^d^	25.89^b^	26.84^c^	27.7^d^
**DSS/LPS-probiotics-amoxicillin**	22.01^b^	20.31^b^	18.36^d^	21.78^b^
**SEM**	1.918	2.429	1.721	1.726
***p*** **value**	< 0.001	< 0.001	< 0.001	< 0.001

Means in the same column, within each parameter, having different superscripts, are significantly different

*TNF-α* tumor necrosis factor-alpha, *IL-1β* interleukin-1β, *IFN-γ* interferon-gamma, *IL-18* interleukin-18, *SEM* standard error of mean

### Effect of Probiotics and Probiotics-Amoxicillin on Caspase-3 Level in the Colon Tissue

**Fig. 2:**
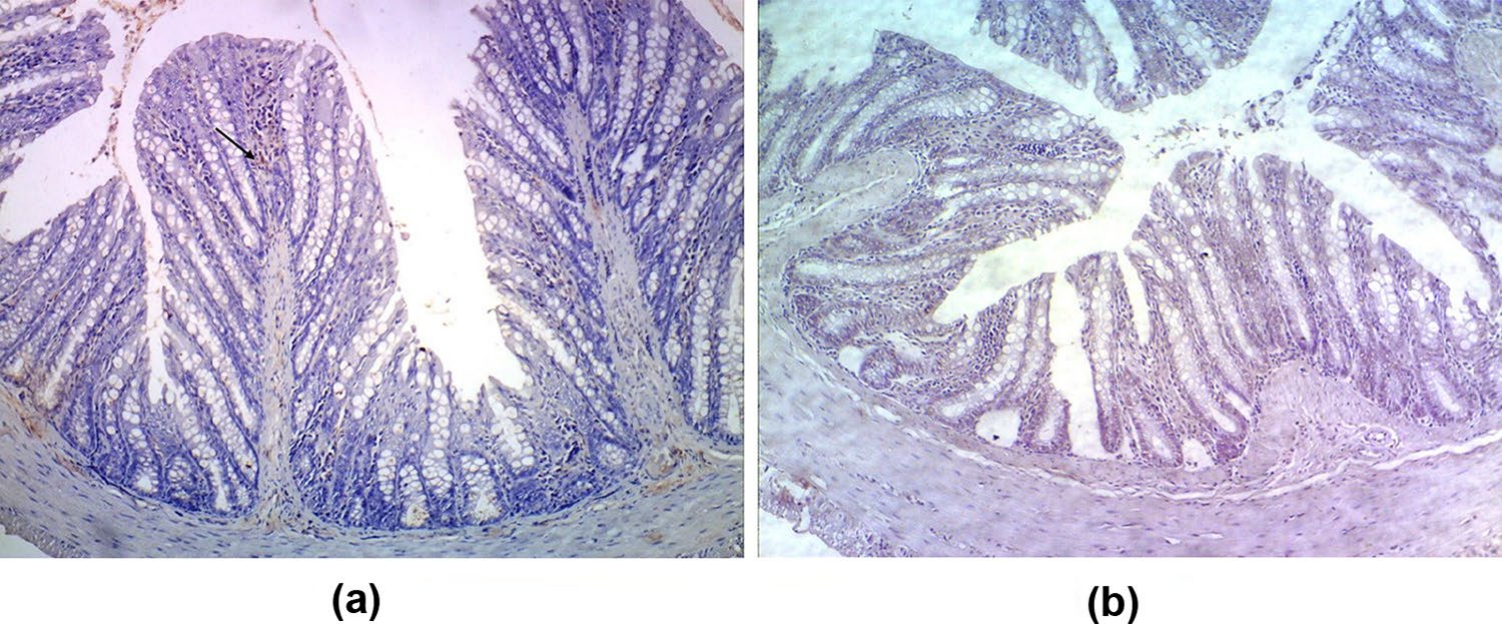
Immunohistochemical staining of caspase-3 in the colon tissue of the **a** control group, **b** probiotics group, showing normal expression of caspase-3 with very few immune-reactive cells (arrows) (× 100)

**Fig. 3:**
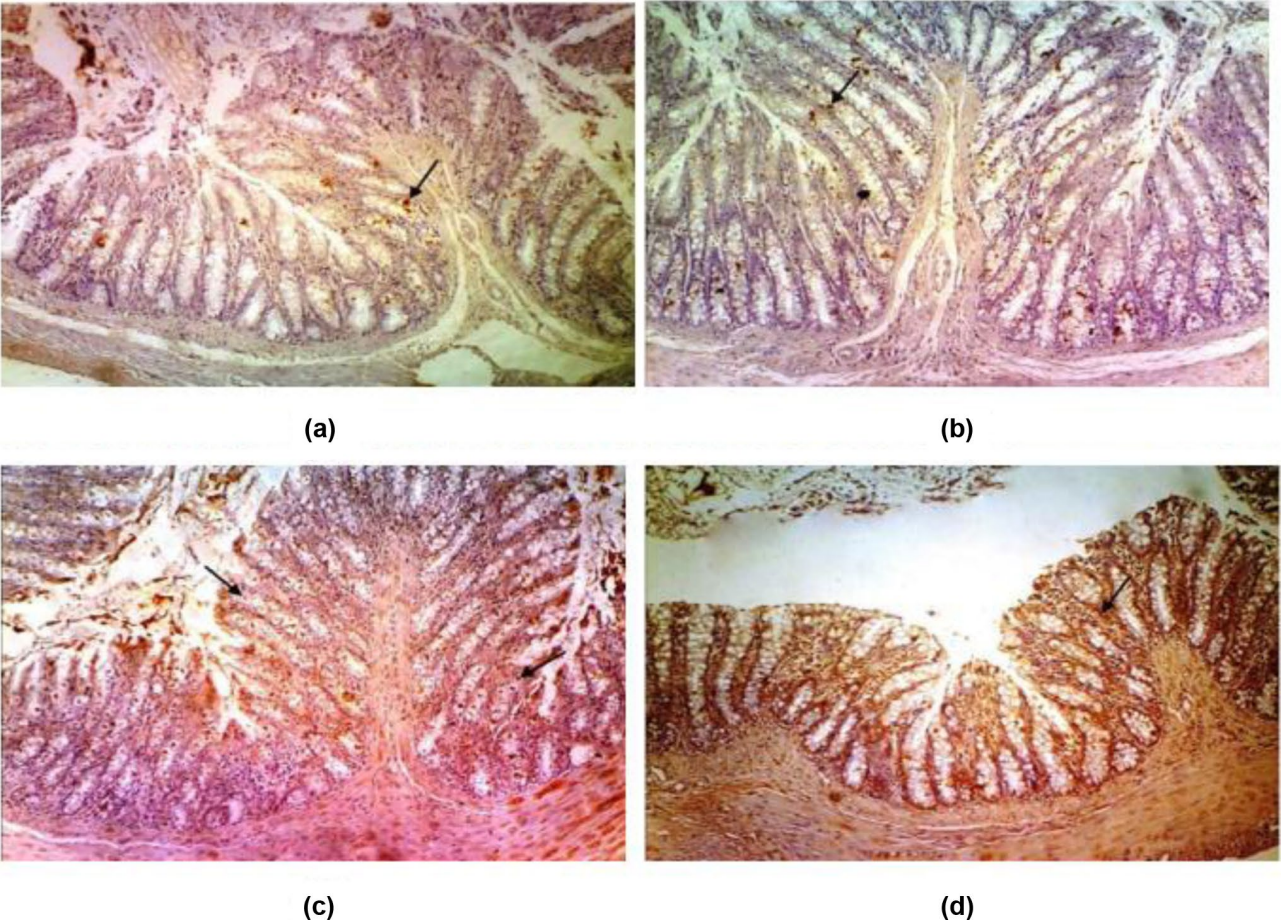
Immunohistochemical staining of caspase-3 in the colon tissue of **a** DSS group, **b** LPS group, showing moderate positive expression of caspase-3 (arrows), **c**, **d** colon tissues of DSS/LPS group showing strong intensely positive expression of caspase-3 (arrows) (× 100)

**Fig. 4:**
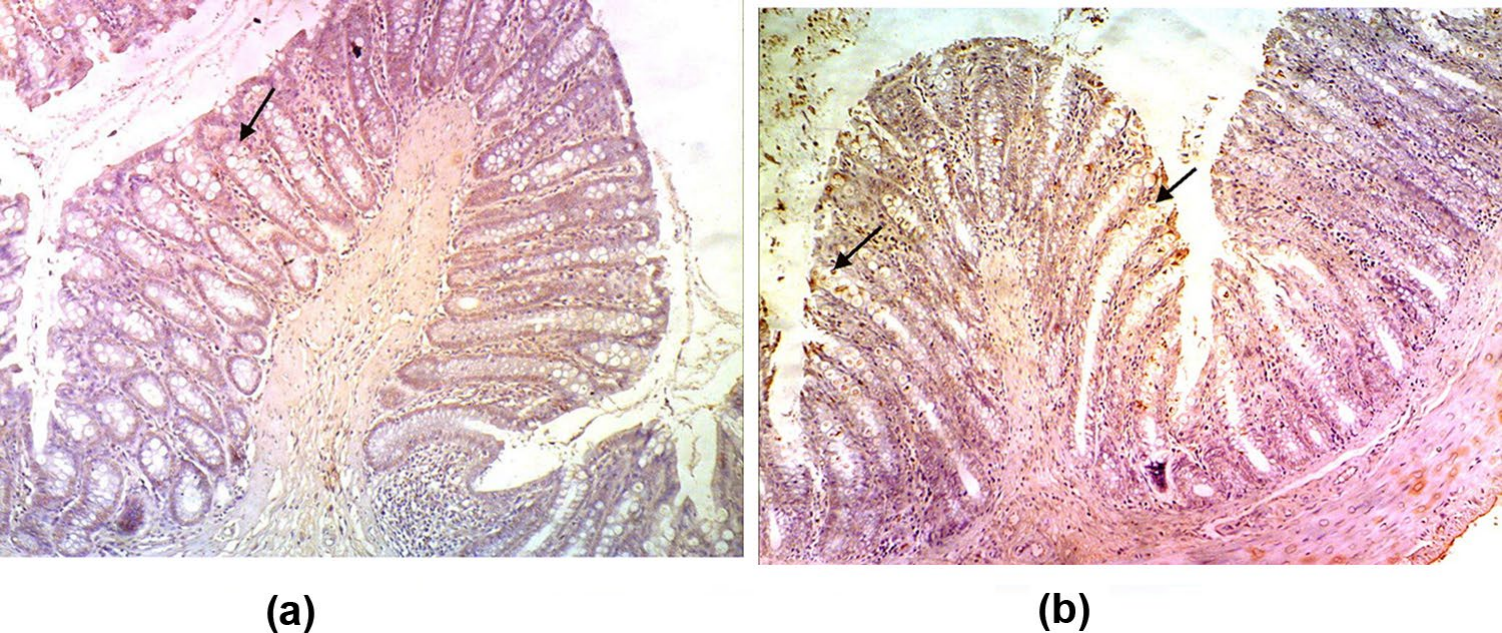
Immunohistochemical staining of caspase-3 in the colon tissue of DSS/LPS treated with **a** probiotics and **b** probiotics-amoxicillin showing moderate positive expression of caspase-3 (arrows) (× 100)

**Fig. 5:**
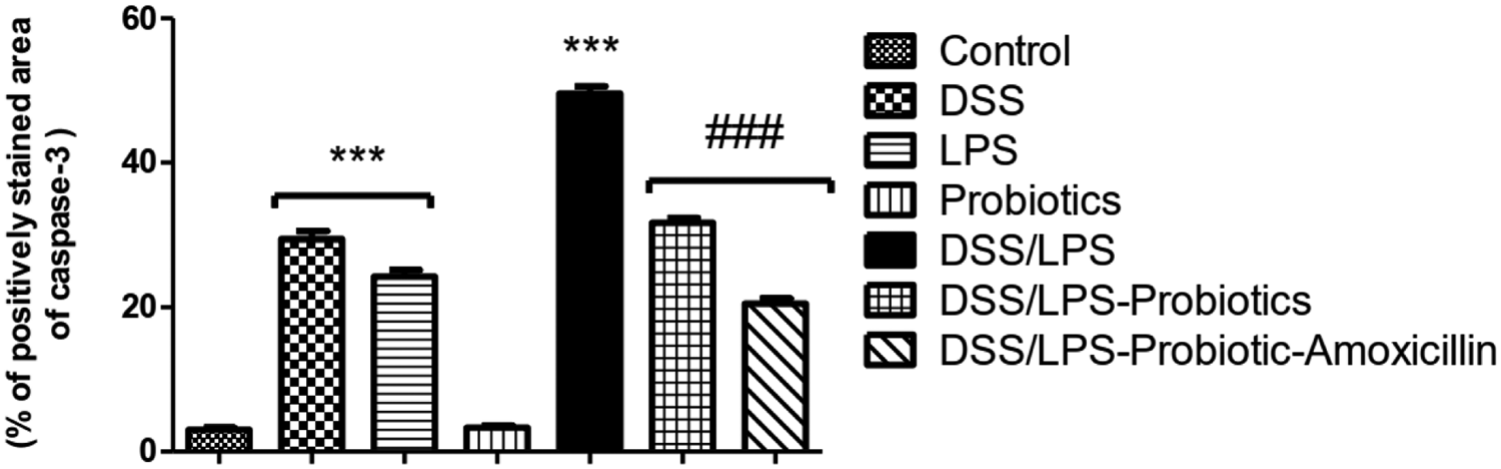
Immunohistochemical signal intensity quantification for caspase-3 expression, in the colon tissue, for the different studied groups. Statistical analysis was performed using one-way ANOVA followed by the Tukey–Kramer test. ^***^ represent significant differences from the control group (^***^p < 0.001). ^###^ represent significant differences from DSS/LPS group (.^###^*p* < 0.001)

The immunohistochemical staining of caspase-3 in the colon tissues of the control group displayed normal expression of caspase-3 with very few immune-reactive cells. Treatment with probiotics maintained the normal expression of caspase-3 (Figs. 2 and 5). Moreover, the colon tissues of DSS and LPS groups showed moderate positive expression of caspase-3 while there was an intensive expression of caspase-3 in the colon tissues of DSS/LPS group (Figs. 3 and 5). On the other hand, treatment with probiotics or probiotics-amoxicillin, in the DSS/LPS manipulated rats, resulted in modulation of caspase-3 expression to the moderate levels (Figs. 4 and 5).

### Effect of Probiotics and Probiotics-Amoxicillin on Aerobic Bacterial Viable Count

**Fig. 6:**
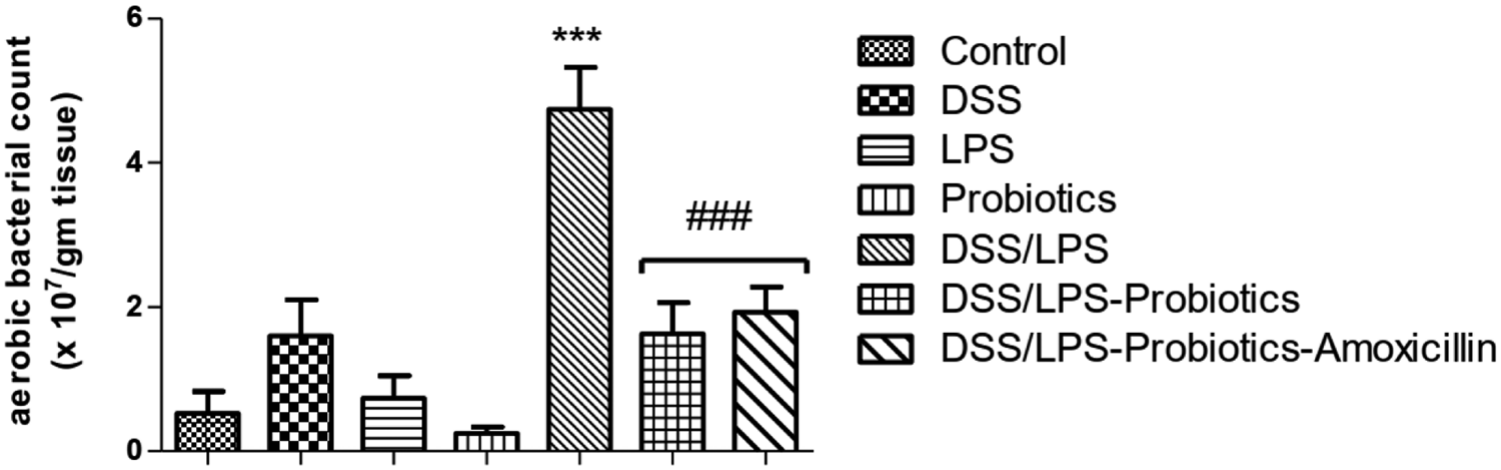
Effect of probiotics and probiotics-amoxicillin on DSS/LPS-induced acute diverticulitis. Aerobic bacterial count, in the colon tissue. Statistical analysis was performed using one-way ANOVA followed by the Tukey–Kramer test. ^***^ represent significant differences from the control group (^***^p < 0.001). ^###^ represent significant differences from DSS/LPS group (^###^*p* < 0.001)

Compared to the control group, DSS or LPS did not show any significant difference (*p* > 0.05) in the bacterial count in the colon tissues. The bacterial count was significantly increased (*p* < 0.001) in DSS/LPS group, compared to the control group. When DSS/LPS-handled rats were treated with probiotics or probiotics-amoxicillin, there was a significant reduction (*p* < 0.001) in the bacterial count, as compared to the DSS/LPS group (Fig. 6).

## Discussion

Colonic diverticular disease is a common acquired condition in which several pathogenic factors may be implicated, including bacterial dysbiosis [23] since the imbalance in the microbial milieu leads to disruption of the immune homeostasis and causes intestinal disorders [24]. The immune responses induced by commensal flora can regulate the composition of the intestinal microbiota, thus maintaining the dynamic balance between commensal bacteria and the host immune system and ensuring gut homeostasis and health [25]. As a result, using antibiotics or probiotics to manipulate the gut microbiota composition has recently been recommended as a therapeutic strategy for acute diverticulitis disease. Antibiotics have long been the cornerstone for the management of acute diverticulitis, intending to prevent inflammation and alleviate associated symptoms [26].

The use of probiotics in diverticular disease has been reviewed by different authors [7, 27]. Probiotics have beneficial effects on the host, since they can enhance the mucosal immune system. Also, they can augment the homeostatic immune defenses as well as restrain infection, inflammation, and allergy [28]. Probiotics accomplish these functions through modulation of the gut microbiome. Their mechanisms of action include the competition with other micro-organisms for nutrients, binding sites, and receptors on the intestinal mucosa. Also, probiotics can inhibit the growth of other microbes through the production of anti-microbial agents [29]. Furthermore, probiotics can reduce pathogen translocation across the intestinal mucosa, by enhancing intestinal barrier integrity and maintaining immune tolerance [30]. They also have been shown to reduce the side effects of the intestinal mucosa by decreasing the levels of anti-inflammatory cytokines and neutrophil infiltration in many studies [31]. *Lactobacillus* and *Bifidobacterium*, both of which have probiotics characteristics, play a pivotal role in the intestinal environment by preventing pathogen colonization and maintaining normal mucosal immunity [32]. Diverticulitis disease and inflammatory bowel disease (Crohn’s disease and ulcerative colitis) have common clinical manifestations, such as abdominal pain, rise of inflammatory markers, and share pathophysiological mechanisms leading to an imbalance between pro-inflammatory and anti-inflammatory cytokines, abnormal colonic motility, and altered intestinal microbiota [27].

In this study, dextran sulfate sodium (DSS) and lipopolysaccharide (LPS) enema were used in the induction of acute diverticulitis disease. DSS induces intestinal inflammation that damages the epithelial monolayer which lines the large intestine, as a consequence; the pro-inflammatory intestinal contents (e.g., bacteria and their products) disseminate into the underlying tissue [33]. According to Zhang et al. [16], killed bacteria induce LPS release, which aggravates colon inflammation.

In the present study, DSS/LPS caused a significant change in the antioxidant status of the colon tissue, since the level of GSH was significantly decreased (*p* < 0.001) while the levels of MDA and NO were significantly increased (*p* < 0.001; *p* < 0.01, respectively). These results are consistent with Rajendiran et al. [34] who reported that GSH content was depleted in the model of DSS-induced colitis. Depletion of GSH leads to the formation of large lymphoid aggregates, in the intestine, through the recruitment of lymphocytes from the peripheral circulation. Also, depletion of tissue GSH has been implicated in the inflammatory process of inflammatory bowel disease (IBD). NO radicals play an important role in the pathogenesis of IBD. Many studies have indicated that excessive concentrations of NO may enhance the progression of ulcerative colitis disease through mechanisms such as direct injury of gut epithelial cells, activation of neutrophils, and vasodilation [35]. MDA is a byproduct of lipid peroxidation. Previous studies have reported that MDA levels increased in ulcerative colitis disease and can be used as an important diagnosis of IBD [36]. On the other hand, the levels of GSH and MDA were restored to the normal values in DSS/LPS-probiotics group and improved in DSS/LPS-probiotics-amoxicillin group. NO levels were retrieved to the normal, only on treatment with probiotics, while its level did not have any significant change on treatment with probiotics-amoxicillin. This improvement in the oxidant/antioxidant status in the colon tissue returns to the antioxidant properties of probiotics, which could be accomplished through; the secretion of glutathione and antioxidant enzymes, direct ROS scavenging action, and their role as strong chelators of free copper and iron ions, to prevent metal ion-catalyzed oxidation. Also, probiotics have been associated with the reduction of the activity of ROS-releasing enzymes, such as NADPH oxidases as well as induction of cellular antioxidant signaling pathways, such as the Nrf2-Keap1-ARE pathway [37].

MPO is an enzyme found predominantly in neutrophils and used as a marker of inflammation and tissue injury [38]. In this study, treatment with probiotics with or without amoxicillin ameliorated the elevation of MPO activity, induced by DSS/LPS. This amelioration is due to the anti-inflammatory properties. The decrease in this enzyme activity reveals a lower infiltration of neutrophils in the inflamed colon tissue since treatment with probiotics caused a reduction in the colonic production of the chemotactic eicosanoid LTB4. It was observed that *Lactobacillus acidophilus* administration reduced colonic MPO activity in the trinitrobenzene sulfonic acid (TNBS) model of rat colitis [39]. Moreover, the inhibitory effect on neutrophil activity resulted in an attenuation of the colonic oxidative stress, as observed in our results, through the restoration of colonic reduced glutathione content after probiotics treatment.

CRP is recognized as one of the most important proteins in acute inflammation [40]. In the present study, DSS/LPS administration led to an elevation in the serum levels of CRP. This elevation was declined on treatment with probiotics or probiotics-amoxicillin due to the inhibition of inflammation and disease progression. It is worth mentioning that higher levels of CRP have been detected in patients with severe acute diverticulitis disease [41]. According to Sartelli et al. [42], CRP has been identified as a useful biomarker of inflammation, and it may be useful in the prediction of the clinical severity of acute diverticulitis.

Regarding the pro-inflammatory cytokines, the current study revealed that DSS and DSS/LPS administration resulted in an elevation of the levels of TNF-α, IL-1β, IFN-γ, and IL-18. Strate and Morris [1] agreed with these findings, demonstrating that many risk factors of diverticulitis are associated with chronic inflammation and alterations in the gut microbiome leading to elevation of the levels of the biomarkers of inflammation. The pro-inflammatory cytokines such as IL-1β and TNF-α were involved in the inflammatory process in DSS-induced colitis [43]. IL-18 is a pro-inflammatory cytokine with stimulatory effects on both T helper-1 and T helper-2 cell responses [44] that have been implied in Crohn’s disease and ulcerative colitis, respectively. IL-18 stimulates the production of IFN-γ and Th1 responses that can promote colitis [45]. Lahat et al. [41] demonstrated that patients with severe acute diverticulitis have more prolonged chronic symptoms, higher inflammatory markers, and higher tissue inflammatory cytokine levels including TNF-α, IL-6, IL-1β, and more inflammatory infiltrates in diverticular colonic tissue. On the other hand, the increased levels of these cytokines were significantly reduced (*p* < 0.001) on either treatment with probiotics or probiotics-amoxicillin. This inhibitory effect is attributed to the existence of a cross-talk between probiotics and mucosal cells. *Bifidobacterium lactis*, either alone or in combination with other probiotics, was able to downregulate the degree of activation of intestinal immune cells [39]. The increased levels of the pro-inflammatory cytokines resulted in uncontrolled apoptosis. These pro-inflammatory cytokines induce apoptosis through suppression of the anti-apoptotic signals in the colon tissue. To evaluate apoptosis in the rats’ colon tissue, paraffin-embedded colon sections were stained for caspase-3 expression by immunohistochemical labeling reaction. The authors observed a significant increase in caspase-3 expression in DSS, LPS, and DSS/LPS groups. On the other hand, the levels of caspase-3 expression were significantly downregulated (*p* < 0.001) with probiotics or probiotics-amoxicillin treatment, compared to DSS/LPS group. Probiotics (*Lactobacillus* spp.) can activate the anti-apoptotic Akt/protein kinase B, as well as inhibit the pro-apoptotic p38/mitogen-activated protein (MAP) kinase [46].

In the present study, the aerobic bacterial count in the descending colon of the control group was 1.5 × 10^7^ CFU/ml. This value agreed with Rambaud [47] who illustrated that gastrointestinal tract aerobic bacteria are mainly streptococci. On the other hand, there was a significant increase in colon mucosa-associated aerobic bacterial count in DSS/LPS group. Lupp et al. [48] agreed with these results demonstrating that host-mediated inflammation in response to infection, chemical agent, or genetic predisposition alters the normal colonic microbial environment due to the augmentation of growth of either the resident or introduced aerobic bacteria, particularly the Enterobacteriaceae family. The current study verified that probiotics with or without amoxicillin could significantly decrease the count of colon mucosa-associated aerobic bacteria. This antimicrobial effect is attributed to the different mechanisms of probiotics in inhibition of adherence or invasion of the pathogenic bacteria [49]. It has been found that treatment with probiotics restored the ratio between pathogenic bacteria and probiotics (*Bifidobacterium lactis* and *Lactobacillus acidophilus*), in the trinitrobenzene sulphonic acid (TNBS) model of rat colitis [39]. Furthermore, Lamiki et al. [9] demonstrated that *Bifidobacterium lactis* and *Lactobacillus acidophilus* showed a protective effect against colon diverticular disease.

Antibiotics have traditionally been used to treat acute diverticulitis in all patients. Recent findings have indicated that the manipulation of antibiotics is not necessary for mild or moderate uncomplicated acute diverticulitis management, as was initially thought [50]. Treatment of acute diverticulitis generally comprises dietary fiber supplementation, anti-inflammatory drugs, pharmacological therapies such as antibiotics, and probiotics, either alone or in combination [51].

In summary, this study indicated that combined probiotics and amoxicillin therapy significantly inhibited acute diverticulitis, exhibiting a protective effect on the colon, which may be related to the inhibition of pro-inflammatory cytokines via IL-1β, TNF-α, IFN-γ, and consequently IL-18 signaling pathway that finally reduces caspase-3 in DSS/LPS-induced acute diverticulitis in the colon tissue. Combined probiotics-amoxicillin therapy was the best at inhibiting the pro-inflammatory parameters used in this study while probiotics therapy, without amoxicillin, was the most effective at restoring the colon tissue antioxidant status. The role of the intestinal milieu, particularly enteric microbiota, seems to be of great significance. In conclusion, our study demonstrates that the administration of probiotics therapy alone or in combination with amoxicillin was able to influence gut microbiota, which suggests its promising treatment option for acute diverticulitis.

## Data Availability

The data used to support the findings of this study are included within this research article. All data generated or analyzed during this study are also included in this published research article.
